# Analysis of Cutting Forces and Geometric Surface Structures in the Milling of NiTi Alloy

**DOI:** 10.3390/ma17020488

**Published:** 2024-01-19

**Authors:** Małgorzata Kowalczyk

**Affiliations:** Chair of Production Engineering, Faculty of Mechanical Engineering, Cracow University of Technology, Jana Pawła II 37 Avenue, 31-864 Krakow, Poland; malgorzata.kowalczyk@pk.edu.pl; Tel.: +48-12-628-3245

**Keywords:** milling, NiTi alloy, cutting force, surface roughness

## Abstract

This paper presents a study of the total cutting force used and selected parameters of the geometric structure of the surface (e.g., *Sa*, *Sz*) during the end milling process of NiTi alloy. The input parameters included are cutting speed (*v_c_*), feed per tooth (*f_z_*), and radial depth of cut (*a_e_*). A Box–Behnken experimental design was employed to conduct the research. The obtained experimental results were utilized within the framework of a response surface methodology (RSM) to develop mathematical and statistical models capable of predicting cutting force components and selected 3D surface parameters. These models provide valuable insights into the relationships between the cutting parameters and the output variables, facilitating the optimization of the NiTi alloy milling process. The findings of this study contribute to a better understanding of the behavior of NiTi alloy during the milling process and offer information for process optimization. By employing a Box–Behnken experimental design, it was possible to investigate the effects of different parameter combinations on the components of total cutting force and selected 3D surface parameters according to ISO 25178, thus aiding in the identification of optimal milling conditions to achieve desired outcomes in the machining of NiTi alloy.

## 1. Introduction

Materials with shape memory and superelasticity are considered new materials. These are original materials with significant potential for applications in modern mechanical structures, materials engineering, or medicine [1,2,3,4,5]. Referred to as multifunctional and intelligent, they are commonly produced as alloys based on nickel and titanium (NiTi), possessing the unique ability to regain their original shape after deformation. The distinct characteristics of the NiTi alloy, coupled with additional advantages such as biocompatibility, high plasticity, a favorable strength-to-weight ratio, corrosion resistance even in aggressive environments, and favorable parameters in terms of mechanical strength, fatigue strength, and specific weight, have fueled a growing interest in materials derived from this alloy in the field of modern mechanical systems utilized in technology and medicine [2,3,4,5,6].

Titanium–nickel alloys, particularly NiTi alloys, are the most extensively researched and practically used shape memory materials, as a result of their exceptionally favorable properties. NiTi alloys exhibit the best shape memory and superelasticity properties and have the most favorable mechanical properties. NiTi alloys are corrosion-resistant materials and due to that property they can be used in aggressive environments, such as seawater, as well as in specific medical applications [1,2]. NiTi-SMAs (shape memory alloys) are also characterized by excellent fatigue properties, high ductility, good forgeability, and high resistance to erosion, abrasion, vibration, etc. [2,5,6].

As the demand for products made of NiTi alloys increases, there is also a need to develop an intelligent machining strategy that will optimize costs and ensure maximum efficiency while maintaining a high quality to the machined surface [7,8,9].

The desired characteristics of these materials heavily depends on the methods used for their production and processing. Therefore, the constant development of new manufacturing technologies and the continuous improvement of existing procedures and processing methods are essential. Due to its high plasticity, temperature sensitivity, and significant surface hardening during machining, the NiTi alloy poses challenges when using conventional manufacturing techniques. The primary issues in machining NiTi alloys are associated with the high temperatures generated during cutting and the rapid wear of the cutting edge, resulting in low cutting process efficiency. Despite the optimization of machining parameters, tool wear still remains a problem in machining of these alloys [5,6]. Continuous chips formed during machining complicate material processing control, and, during that process, burrs are generated, contributing to the low quality of the machined surface [7,10,11,12]. The material’s high strength also induces additional mechanical loads at the tool–workpiece interface, leading to strong adhesion on the tool flank face [13,14,15,16]. The increased adhesion of the workpiece material to the cutting tool with prolonged machining periods is observed as an unfavorable phenomenon [17,18].

The milling process is widely employed in most manufacturing process chains for machining materials such as metals, titanium alloys, nickel alloys, ceramics, and composites. It allows greater dimensional accuracy, a finer surface finish, complex shapes, and a wide range of manufactured component sizes. Therefore, milling processes play a crucial role in producing complex components made of shape memory alloys, such as the NiTi alloy [18,19,20,21]. However, milling this alloy is challenging due to its material properties, which include high hardness, low thermal conductivity, and poor machinability [14,15,18].

In comparison to other engineering materials, the machinability of NiTi is undeniably a greater challenge. Challenges related to the machining of these alloys hinder the achieving of higher cutting parameters, thus increasing machining efficiency while maintaining the required quality of the surface layer [19,20,21,22,23]. The unique properties of NiTi alloys, such as shape memory and superelasticity, introduce complexities into the machining process that set them apart from conventional engineering materials. It is worth noting that, compared to titanium alloy, nickel alloy, maraging steel, etc., machining a NiTi alloy may present even greater challenges due to the unique properties of the alloy [5,13,24]. These distinctive characteristics necessitate specialized machining approaches and tooling strategies to address the inherent challenges and optimize the machining of NiTi alloys [13,25]. However, to uphold the importance of NiTi alloys as construction materials, efforts should be directed towards reducing their processing costs (due to their challenging machinability). Historically, the industry’s priority has been obtaining high-quality parameters for products made of the NiTi alloy, with production cost as a secondary concern. It can be anticipated that, in the future, due to the need to reduce and optimize production costs, research efforts will be focused on shaping NiTi alloy elements using methods that ensure maximum efficiency while maintaining the quality of the machined surface [19,21,23,24,25,26].

The growing use of shape memory alloys like the NiTi alloy, in various industries, and the demand for processing such materials result in the continuous development of machining technology. This includes the design of tools, the use of new tool materials, and the rational selection of cutting parameters, as well as research into phenomena occurring during machining and their alignment with real conditions [9,27,28].

The fragmentary experimental results presented in the scientific literature are insufficient for developing guidelines for the processing of NiTi alloys. The geometric structure of the surface formed during milling depends not only on the kinematic and geometric parameters of the process but also on the tool displacements caused by the impact of the forces and geometric errors of the elements of the M–H–W–T (Machining Operation–Holder–Workpiece–Tool) system. Most studies analyzing the surface formed during milling do not consider the combined impact of the aforementioned factors [8,29,30].

Therefore, a detailed analysis of the cutting force and the geometric structure of the surface during the milling process of NiTi alloy can provide important insights for planning this process. This is particularly crucial when producing shape memory materials that are challenging to process. By analyzing the interplay between cutting parameters and surface characteristics, this analysis aims to unravel new insights that can pave the way for enhanced machining strategies. The integration of advanced experimental designs, such as the Box–Behnken method, allows to delve into unexplored dimensions, offering a comprehensive understanding of the milling dynamics specific to NiTi alloys. The conducted research will contribute to understanding the milling process of NiTi alloys and provide valuable insights for optimizing milling parameters. The results could help manufacturers improve machining efficiency and achieve a high-quality surface finish after milling a NiTi alloy, ultimately facilitating the widespread use of this advanced material in various engineering applications.

This endeavor aspires to shed light on the untapped potential within the intricate realm of NiTi alloy machining, shaping the trajectory of future advancements in materials processing.

## 2. Research on Stand Components and Tested Materials

**Figure 1:**
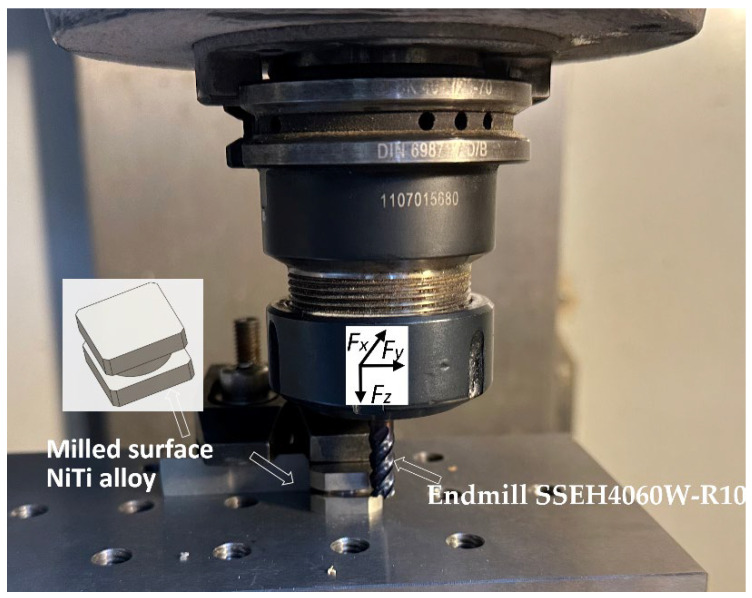
Experimental set up for milling NiTi.

The cutting process was examined on a test stand at the Chair of Production Engineering (Cracow, Poland) on a CNC milling machine tool Haas VF1 equipped with a digital control unit. The experimental setup is illustrated in Figure 1 (milled-surface NiTi alloy and cutting tool).

The 3D topography measurements of the treated surface were made using the Taylor Hobson measuring system. The TalyMap program was used to visualize surface test measurements. In this study, measurements of the surface topography of the selected parameters were made under the following conditions, which are shown in Table 1. In addition, a Gaussian filter was applied. The measurements were made in triplicate for statistical purposes. The post-processing of the raw data included noise removal, shape profile filtering, topography imaging with 3D maps, the determination of selected surface topography parameters, and their statistical evaluation. The parameters of the surface topography were determined following ISO 25178 [7,9].

A workpiece is usually employed in the aerospace industry to manufacture components. The material used in this work was NiTi shape memory alloy, which was slightly off-stoichiometry, with 57.88 Ni (wt.%), obtained from Baoji Hanz Metal Material Co. Ltd. (Baoji, China). The austenite finish temperature was *A_f_* = 60 °C. Table 2 shows the chemical composition (wt. [%] and at. [%]) of the NiTi alloy. The physical, thermal, and mechanical properties of the β-NiTi of the material are shown in Table 3, respectively.

**Figure 2:**
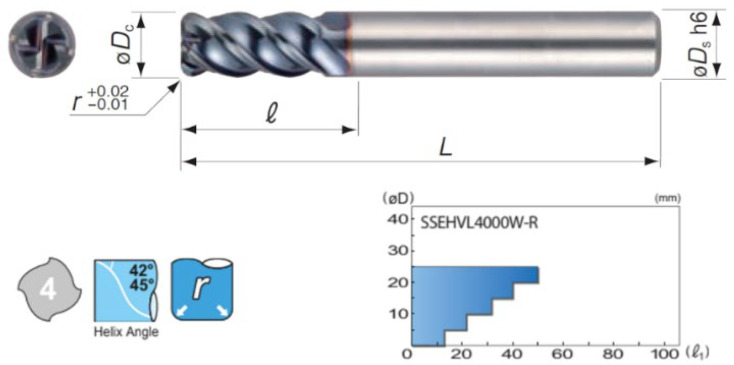
Coated carbide end mill (grade: ACW52) [Sumitomo].

Dry down milling was used in the experiments. The cutting tool was a 6 mm diameter coated carbide end mill (grade: ACW52) with four teeth (Sumitomo SSEH 4060W-R10), presented in Figure 2.

The research plan to determine the impact of three independent factors, namely, feed per tooth ($f_{z}\;[mm/tooth]$), radial depth of cut ($a_{e}$ [mm]), and cutting speed ($v_{c}$ [m/min]), on the values of the selected factors Sa and Sz (i.e., 3D areal surface texture parameters), of the surface roughness and cutting force components, was developed according to Response Surface Design [9,26]. The Box–Behnken experimental design approach was adopted during this research.

The Box–Behnken experimental design employed in this study serves as a pivotal methodological framework for systematically exploring the effects of various factors on the milling process of NiTi alloy. Developed by George E.P. Box and Donald W. Behnken, this design is a response surface methodology that efficiently balances the number of experimental runs required with the precision of the obtained results. The Box–Behnken design facilitates the creation of a set of experimental combinations, or “runs”, that allows us to model and analyze the behavior of the milling process within this multi-dimensional parameter space. The key advantage of the Box–Behnken design lies in its ability to generate a quadratic model that captures both the linear and interaction effects of the chosen factors. This is particularly advantageous when studying complex machining processes, such as the end milling of NiTi alloy, where the relationship between input parameters and output responses may exhibit non-linear behavior. The design achieves efficiency by utilizing fewer experimental runs compared to a full factorial design while maintaining the capability to discern the main effects and interactions. Each run in a Box–Behnken design represents a unique combination of factor levels, and the resulting data are then utilized to fit a mathematical model. This model, often in the form of a polynomial equation, allows for the prediction of responses across the entire experimental domain [31,32].

The order of runs was generated by the Minitab program (Table 4). The range of variability of the input quantities included:

✓ A cutting speed *v_c_* in the range of 30 ÷ 50 m/min;
✓ The feed per tooth *f_z_* in the range of 0.01 ÷ 0.04 mm/tooth;
✓ A radial depth of cut *a_e_* in the range of 0.2 ÷ 0.4 mm;
✓ A constant axial depth of cut *a_p_* = 4.00 mm.

To select cutting parameters for the study, a combination of literature review and pilot experiments was adopted. On this basis, it was decided that the cutting speed should be set between 30 and 50 m/min, the feed per tooth between 0.01 and 0.04 mm/tooth, and the radial depth of cut between 0.2 and 0.4 mm. The experimental design matrix is provided in Table 4.

The factors Sa (i.e., the arithmetical mean height of the surface) and Sz (i.e., the maximum height of the surface—the sum of the maximum peak height value and the maximum pit depth value within a defined area) were defined as per the ISO 25178 standard [7,9].

Generally, Sa is represented by the equation [7,9]

(1) $$Sa=\frac{1}{LB}\int_{0}^{L}\int_{0}^{B}\left|\eta (x,y)\right|dxdy$$

where $\eta \left(x,y\right)$ is the deviation of the surface irregularities from the base plane, and L and B are the length and the width of the given section of the surface corresponding to the baseline for the given type of surface irregularities, respectively.

Surface quality, as measured by the roughness index during machining, primarily stems from process characteristics, including cutting tool geometry (e.g., corner radius and rake angle), cutting parameters (e.g., cutting speed, feed, and depth of cut), tool wear, cooling methods, and the material properties of both the tool and the workpiece [9,21,22]. Surface roughness serves as a vital indicator of product quality and significantly impacts production costs. It delineates the surface geometry to be processed and, in conjunction with surface texture, which varies with the machining process, can profoundly influence tool performance (e.g., the appearance of excessive friction and wear). Obtaining the desired roughness value is an iterative and empirical process that can be time-consuming, often requiring multiple attempts until an acceptable value is attained. Since identifying the mechanism of surface roughness formation is complex and process-dependent, it is challenging to calculate using analytical formulas. Hence, precise analysis is essential to assess the surface roughness during the machining of NiTi alloy [9,17,33].

Furthermore, cutting force components, which affect both the tool and the workpiece, induce changes in the tool’s position relative to the workpiece. Any deviation occurring during machining can negatively impact the dimensional accuracy of the machined surface. Analyzing and optimizing cutting forces before actual machining can provide crucial insights for planning the milling process, especially when dealing with challenging-to-machine NiTi alloys [27,29,30].

**Figure 3:**
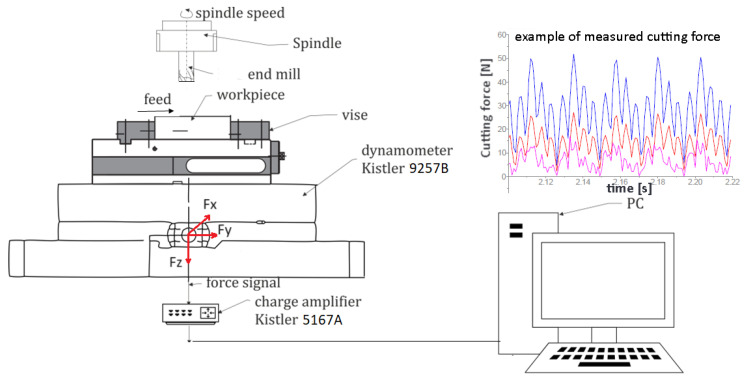
Schematic representation of the measurement of the cutting force.

**Figure 4:**
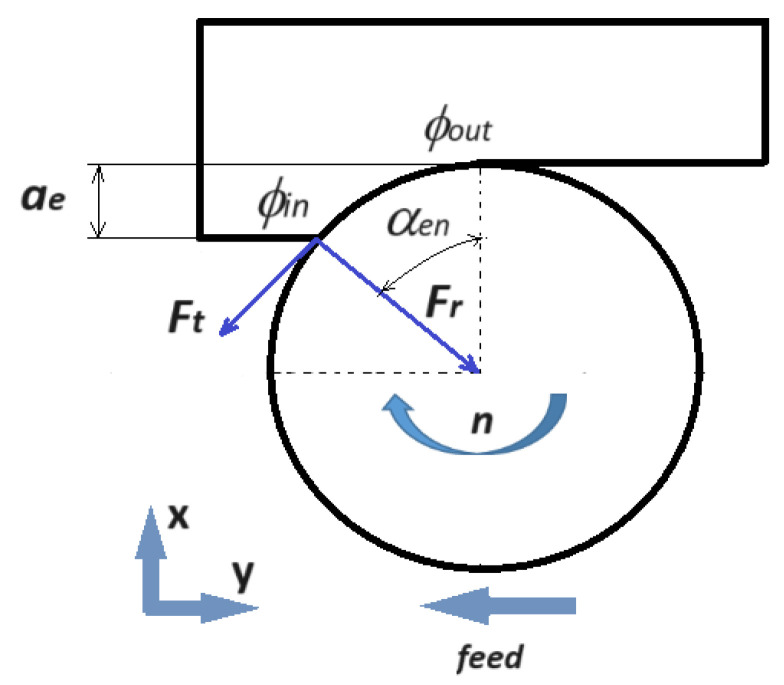
Schematic representation of the cutting force component in the tool system, where $a_{e}$ [mm]—the radial depth of cut, $\emptyset_{in}$ [rad]—entry angle, $\emptyset_{out}$ [rad]—exit angle, $\alpha_{en}$ [rad]—radial engagement angle, n [rev/min]—spindle speed, $F_{t}$ [N]—tangential cutting force, and $F_{r}$ [N]—radial cutting force [34].

The measuring system consists of a Kistler 9257B piezoelectric dynamometer, which enables the measurement of the cutting force components in the machine tool system: the thrust (axial) force component (*F_z_*), the feed force component (*F_y_* = *F_f_*), and the force in the X direction component (*F_x_*). The data were acquired using data acquisition software (DynoWare.exe Version 3.1.2.0), and observations were tabulated to obtain a mathematical model. A schematic representation of the experimental setup is shown in Figure 3. The obtained results for the cutting force represent an average of 3 measured values. The relationship between cutting forces (tangential force *F_t_*, radial force *F*_r_, and feed force *F_y_*= *F_f_*) and cutting parameters was analyzed. A schematic representation of the cutting force component of the tool system is shown in Figure 4.

The analysis of forces in the milling process provides information on the mechanics of the cutting process, taking into account the properties of the workpiece material and tool geometry. To model the cutting forces for end milling, a mechanistic force model is applied, which has been proven in previous research [34,35,36]. This model relates the cutting force components to the undeformed chip thickness. Tangential (*F_t_*) and radial (*F_r_*) forces are applied on the kth flute of a rigid end mill at time *t*. The tangential and radial forces are then resolved into feed (*F_y_*) and transverse (*F_x_*) force components as

(2) $$F_{t}=F_{y}cos\alpha_{en}+F_{x}sin\alpha_{en}$$

(3) $${\;F}_{r}=F_{x}cos\alpha_{en}{-F}_{y}sin\alpha_{en}$$

In down milling, for a single fluted endmill, the shape of the cutting force depends on both the cutter engagement angle $\alpha_{en}$ and the cutter swept angle $\alpha_{sw}$. These angles are related to the cutting parameters [34].

The engagement angle $\alpha_{en}$ is defined using the radial depth of cut *a_e_* in the following equation:

(4) $$\alpha_{en}=\emptyset_{out}-\emptyset_{in}=a\cos(1-\frac{a_{e}}{D})$$

The axial engagement angle $\alpha_{sw}$ is related to the axial depth of cut *a_p_* through the following equation:

(5) $$\alpha_{sw}=\frac{2\tan(\alpha_{el})}{D}a_{p}$$

where *a_e_* is the radial depth of cut, *a_p_* is the axial depth of cut, *D* is the tool’s diameter, and $\alpha_{el}=45^{o}$ is the cutter’s helix angle parameters.

Indeed, $the\;angle\;\theta =\emptyset_{in}+\alpha_{sw}\;$ represents angular positions where the cutting edge is fully involved in the cut, thus identifying a maximum cutting force [34].

## 3. Results and Discussion

**Figure 5:**
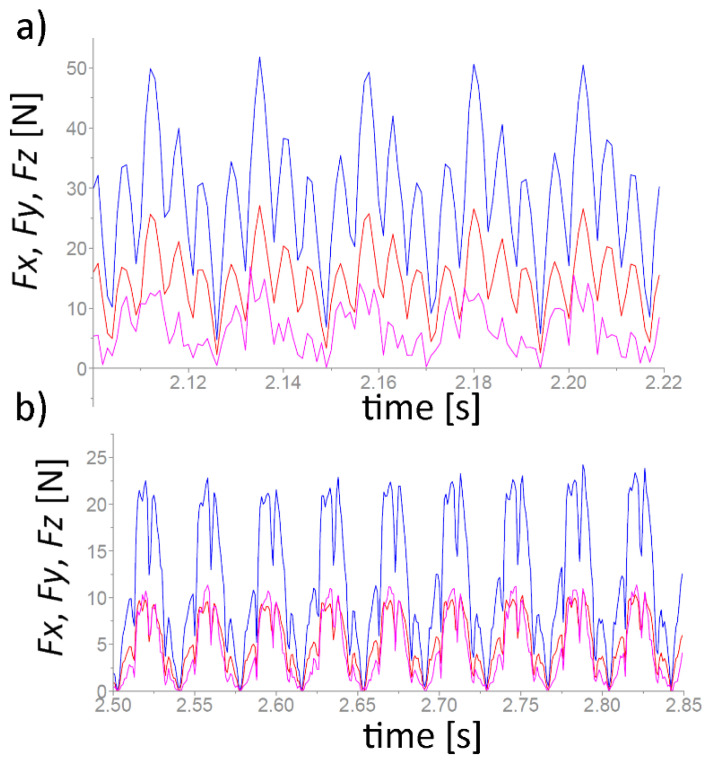
The result of cutting force components for different kinds of feed per tooth *f_z_*; test 11 (**a**) and test 7 (**b**), where *F_x_*—blue color, *F_y_*—red color, and *F_z_*—pink color.

Table 5 presents the results of measurements of the maximum cutting force components during the milling of a NiTi alloy for the adopted research range. Figure 5 shows the results of cutting force components for different kinds of feed per tooth *f_z_*; test 11, where *v_c_* = 30 m/min, *f_z_* = 0.04 mm/teeth, and *a_e_* = 0.3 mm (a), and test 7, where *v_c_* = 30 m/min, *f_z_* = 0.01 mm/teeth, and *a_e_* = 0.3 mm (b).

The statistical analysis used to determine the response surface methodology was used to model the relationship between the parameters of the cutting process and the components of the cutting forces, as well as the parameters of the surface topography. The response surface methodology (RSM) represents a powerful amalgamation of mathematical and statistical techniques. It involves fitting a curved surface based on experimental test data to accurately simulate the real curved surface under specific conditions, facilitating a reliable analysis of the results. The multivariate quadratic regression method is employed as a tool for determining dependencies. By capturing the interactions of all factors and test outcomes, a polynomial equation is established that precisely characterizes the relationship between the factors and the response values. Extracting optimal parameter values from the response surface aids in effectively minimizing research costs while ensuring optimal outcomes [9,25].

Based on the response surface methodology, appropriate models were selected and adequate estimated regression coefficient equations were obtained for the values of the *F_f max_*, *F_r max_*, and *F_t max_* force components for a single flute (6)–(8).

(6) $$F_{rmax}=-47.32+0.20v_{c}+1518.05f_{z}+173.62a_{e}$$

(7) $$F_{fmax}=-29.86+0.14v_{c}+1251.92f_{z}+101.77a_{e}$$

(8) $$F_{tmax}=-9.59+0.33v_{c}+1134.92f_{z}+82.38a_{e}$$

A multifactorial analysis of variance (ANOVA) was conducted for the maximum values of the cutting force components, *F_rmax_, F_tmax_*, and *F_fmax_*, to determine the influence of cutting factors (independent variables) on the dependent variable (Table 6). The dependent variable comprised the values of individual force components, while the independent variables were the radial depth of cut (*a_e_*), feed per tooth (*f_z_*), and cutting speed (*v_c_*). One of the fundamental aspects in the analysis of variance involves estimating the so-called mean square (MS). The ratio of mean squares calculated for each factor to the mean square error allowed for the assessment of the individual impact of each factor on the level of the dependent variables.

The analysis of variance (ANOVA) results for the maximum cutting force components (*F_rmax_*, *F_fmax_*, *F_tmax_*) revealed the significant effects of the considered cutting parameters (*f_z_*, *a_e_*) on the dependent variables. For *F_rmax_*, both factors *f_z_* and ae demonstrated statistically significant effects, with p-values less than 0.05, indicating their influence on the total cutting force. In contrast, the cutting speed (*v_c_*) did not exhibit a significant effect on *F_rmax_*, suggesting its limited impact in this context. Similarly, for *F_fmax_* and *F_tmax_*, the cutting parameters *f_z_* and ae exhibited significant effects, while cutting speed (*v_c_*) did not demonstrate a substantial influence. The lack-of-fit tests indicated that the models adequately represented the data, with p-values greater than 0.05, affirming the appropriateness of the chosen models. These findings underscore the nuanced influence of specific cutting parameters, emphasizing the need for meticulous consideration and optimization to achieve the desired outcomes from the milling process of NiTi alloy.

**Figure 6:**
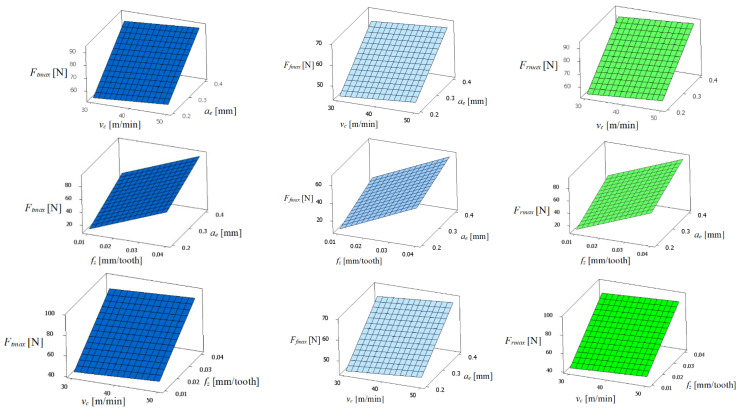
Graph of the components of cutting forces, *F_tmax_, F_fmax_*, and *F_rmax_,* for a single flute.

Figure 6 shows the graphs of the components of the cutting forces as a function of the cutting speed (*v_c_*), feed per tooth (*f_z_*), and radial depth of cut (*a_e_*). The analysis of Figure 6 shows that the cutting force components for a single flute have a very similar course. Both the feed force component (*F_f_*), tangential force component (*F_t_*), and radial force component (*F_r_*) practically do not change with the increasing cutting speed (*v_c_*). Their increase occurs with the increase in feed per tooth (*f_z_*) and also the radial depth of cut (*a_e_*). Incrementing the feed per tooth from *f_z_* = 0.01 mm/tooth to *f_z_* = 0.04 mm/tooth increases the feed force component (*F_f_*) by about 300%, but the tangential force component (*F_t_*) and radial force component (*F_r_*) by about 200%. It should be noted that the feed force component (*F_f_*), the thrust force component (*F_p_*), and the radial force component (*F_r_*) are strongly correlated with each other.

Based on the final milling tests of the NiTi alloy with the use of a coated carbide end mill, the influence of the cutting parameters on the selected parameters of the surface topography was determined. Table 7 presents the results of measurements of the surface topography parameters after the milling process.

Based on the response surface methodology, appropriate models were selected and adequate estimated regression coefficient equations were obtained for the values of the *Sa* and *Sz* parameters of the surface topography (9), (10).

(9) $$Sa=7.287-0.193v_{c}-2.838f_{z}-20.055a_{e}+0.001v_{c}^{2}-689.074f_{z}^{2}+9.321a_{e}^{2}+0.858v_{c}f_{z}+0.385v_{c}a_{e}+15f_{z}a_{e}$$

(10) $$Sz=200.67-5.5v_{c}-2229.19f_{z}-360.12a_{e}+0.03v_{c}^{2}+7003.70f_{z}^{2}+128.08a_{e}^{2}+42.23v_{c}f_{z}+7.03v_{c}a_{e}+306.67f_{z}a_{e}$$

**Figure 7:**
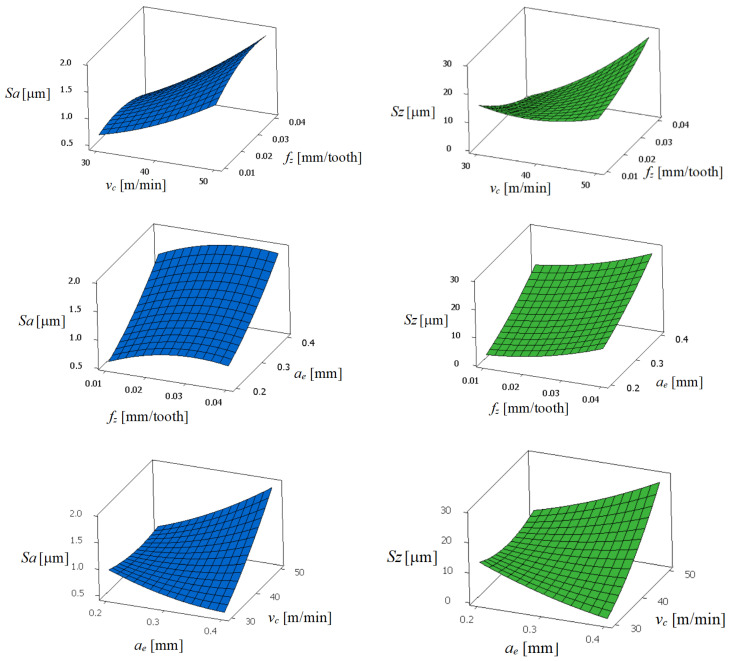
Graph of the surface roughness parameters *Sa* and *Sz*.

Figure 7 shows the graphs of the parameters of the surface roughness, *Sa* and *Sz*, as a function of the cutting speed (*v_c_*), the feed per tooth (*f_z_*), and the radial depth of cut (*a_e_*). The analysis of Figure 7 shows the variation of surface roughness, *Sa* and *Sz*, with respect to the process factors. The parameter of surface roughness *Sa* increases with increases in the cutting speed (*v_c_*) and the radial depth of cut (*a_e_*), however, the influence of the feed per tooth on roughness is not as significant as the two other parameters. For a feed per tooth *f_z_* = 0.025 mm/tooth, cutting speed *v_c_* = 50 m/min, and a radial depth of cut *a_e_* = 0.4 mm, the surface roughness parameters *Sa* and *Sz* are the highest, and their values are *Sa* = 2.13 μm and *Sz* = 25.8 μm. Therefore, the optimal combination of cutting conditions for the surface roughness parameters *Sa* and *Sz* within the testing area for the down milling should be a maximum feed per tooth *f_z_* = 0.04 mm/tooth, a radial depth of cut *a_e_* = 0.3 mm, and a maximum cutting speed *v_c_* = 30 m/min.

The surface condition of the milled NiTi alloy was then subjected to qualitative and quantitative analysis. The characteristics of the surface topography after milling with different cutting parameters are presented in Table 8 (isometric image and selected surface parameters), and were obtained using a profilometer.

According to the presented results in Table 8, it is seen that as the process factors increase from their initial range to a middle range, i.e., (feed per tooth from 0.01 to 0.025 mm/tooth, the cutting speed from 30 to 40 m/min, and the radial depth of cut from 0.2 to 0.3 mm), the magnitude of the roughness indices *Sa*, *Sz*, *Sq*, *Sp*, and *Sv* decrease (e.g., tests no. 7, 9, 13, 15, and 7–11). On the other hand, by further increasing the process factors from their middle range to their maximum one, it is inferred that the amplitude of roughness indices are increased (e.g., tests no. 3–9, 3–11, and 4–10). The remaining amplitude parameters *Ssk* and *Sku* provided additional information on the geometric structure of the milled surfaces. Both the skewness coefficient *Ssk* and the concentration coefficient *Sku* are sensitive to valleys and peaks (including defects) occurring on the surfaces. The *Ssk* parameter for surfaces 4, 7–10, and 15 had a negative value, which indicated a plateau shape to these surfaces, compared, for example, to surfaces 2 and 3. The lower the value of the *Ssk* parameter, the more flattened the surface and the more rounded the tops of the peaks. From the point of view of the interaction of the surfaces in terms of friction node elements, the surfaces characterized by wide peaks with rounded tops are considered to be favorably shaped, while the surfaces characterized by narrow hills with sharpened tops are considered to be unfavorably shaped. In the case of the tested surfaces, surface no. 11 was preferably shaped. The *Sku* parameter (with a value oscillating around 3) revealed that the tested surfaces are characterized by a distribution of ordinates close to the normal distribution (Table 8—Abbott’s curve), which means an even distribution of both peaks and valleys on the surfaces of the tested samples (for example, surfaces 8, 10, 11, and 15).

**Figure 8:**
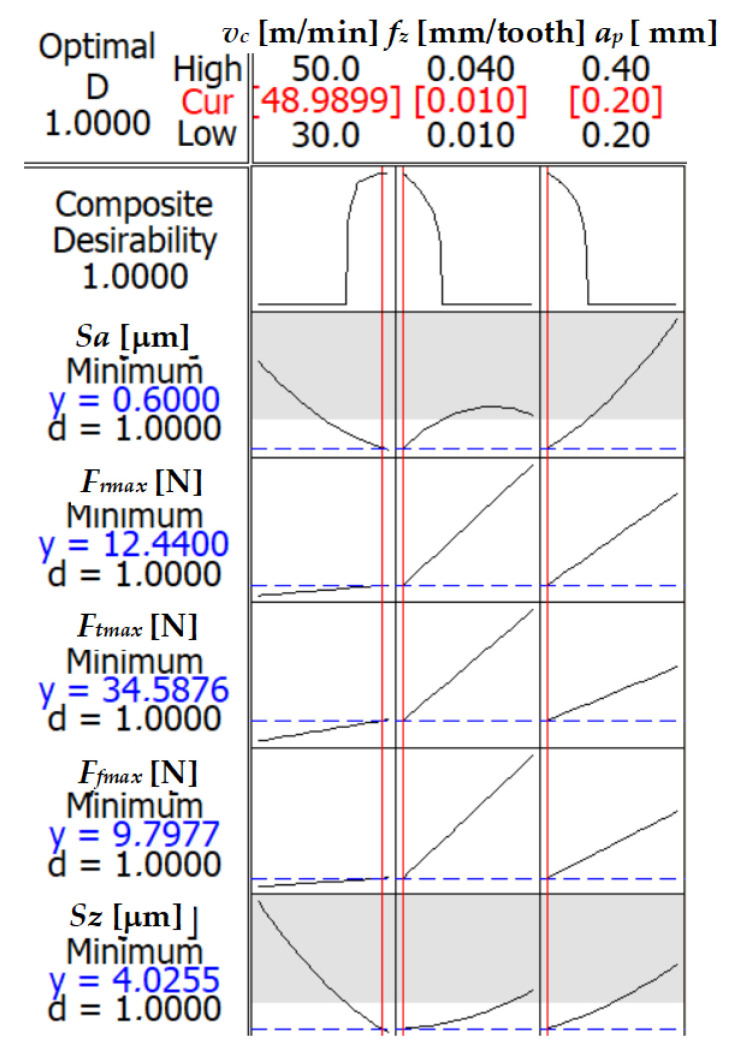
Multi-criteria optimization for cutting force components and surface roughness parameters *Sa* and *Sz*—RSM.

An optimization process was conducted to provide recommendations for cutting parameters that effectively address both the cutting forces and the *Sa* parameter of surface roughness. The refined parameters aim to achieve a harmonious balance, minimizing cutting forces while concurrently optimizing surface roughness. This optimization reflects a commitment to delivering comprehensive recommendations for the machining process. The optimization plot for the components of the cutting forces is shown in Figure 8, using the response surface methodology (RSM).

In the pursuit of minimizing the response surface methodology (RSM) process, a multi-criteria optimization was conducted (Figure 8). The optimized input parameters were determined as follows: cutting speed (*v_c_*) at 50 m/min, feed per tooth (*f_z_*) set to 0.01 mm, and a radial depth of cut (*a_e_*) equal to 0.2 mm. The corresponding output parameters achieved through this optimization were a surface roughness (*Sa*) of 0.6 μm, roughness depth (*Sz*) of 4.03 μm, radial cutting force (*F_rmax_*) of 12.24 N, tangential cutting force (*F_tmax_*) of 34.59 N, and feed cutting force (*F_fmax_*) of 9.78 N. The composite desirability index reached an optimal value of 1.00.

These results indicate that the selected combination of cutting parameters leads to the desired outcome, characterized by minimized surface roughness and cutting forces. The achieved composite desirability of 1.00 suggests an excellent agreement with the optimization goals, emphasizing the effectiveness of the applied approach in obtaining optimal machining conditions.

## 4. Conclusions

Based on the conducted research, it can be concluded that the milling of a NiTi alloy poses significant challenges. The unidentified occurrence of peaks and depressions impacts the roughness parameters, with their nature and distribution being random. This randomness may suggest their origin as a by-product of the growth associated with the processing of the NiTi material.

Modern technology, instrumentation, and software now enable the swift and automated determination of 3D surface roughness parameters, the generation of a material fraction curve, and the analysis of 3D surface topography. This capability allows for the identification of optimal conditions for the cutting process to achieve optimal properties of the machined surface.

The obtained results facilitate the determination of the machining parameters required, such as feed per tooth (*f_z_*), radial depth of cut (*a_e_*), and cutting speed (*v_c_*), for achieving the expected surface roughness during machining. The analysis reveals that the parameters of surface roughness follow a similar trend. Both parameters *Sa* and *Sz* of surface roughness increase with a rise in the cutting speed (*v_c_*) and the radial depth of cut (*a_e_*). The lowest values of the roughness parameters for surfaces machined from the NiTi alloy were achieved with the following cutting parameters: *v_c_* = 30 m/min; *f_z_* = 0.04 mm/tooth; and *a_p_* = 0.3 mm, while the highest values were obtained with the following parameters: *v_c_* = 50 m/min; *f_z_* = 0.025 mm/tooth; and *a_p_* = 0.4 mm.

Additionally, measuring the cutting force components during the milling process of a NiTi alloy is crucial for process evaluation. The analysis reveals that the cutting force components follow a similar trend. Both the feed force component (*F_f_*), tangential force component (*F_t_*), and radial force component (*F_r_*) exhibit minimal changes with increasing cutting speed (*v_c_*). Their increase is associated with a greater feed per tooth (*f_z_*) and radial depth of cut (*a_e_*). The lowest values for the total cutting force components were recorded when machining the NiTi alloy with the following cutting parameters: *v_c_* = 40 m/min; *f_z_* = 0.01 mm/tooth; and *a_p_* = 0.2 mm, while the highest values were obtained with the following parameters: *v_c_* = 40 m/min; *f_z_* = 0.04 mm/tooth; and *a_p_* = 0.4 mm.

In summary, measurements of the cutting force components and the qualitative and quantitative analysis of parameters characterizing the geometric structure of the tested surfaces enable the definition and planning of further steps to improve the milling process of challenging-to-cut materials, including the NiTi shape memory alloy. The appropriate selection of cutting parameters allows for the obtaining of surfaces with the required technological quality.

Furthermore, the multi-criteria optimization conducted in this study demonstrates a balance between cutting forces and surface roughness. The application of innovative optimization algorithms and methodologies contributes to the advancement of the precision machining of NiTi alloys, ensuring that the selected combination of cutting parameters leads to the desired outcome, with a composite desirability index reaching an optimal value of 1.00.

The primary emphasis on innovation and novelty in this article lies in the comprehensive analysis of 3D surface roughness parameters. By delving deeply into the intricate details of the 3D surface’s characteristics, an approach has been introduced to help understand and optimize the milling process of NiTi alloys. This comprehensive examination of surface topography parameters using advanced technology, instrumentation, and software distinguishes this study from conventional approaches, demonstrating a forward-looking perspective in the field of machining NiTi alloys.

## Figures and Tables

**Table 1:** The selected parameters for measurement of the milled surface of *β*-NiTi alloy $A_{f}=60\;℃$.

Sampling Length (Cut-Off, *λ*_c_)	Evaluation Length, ln	Number of Sections	Registered *Nx*	Sampling Step, Δ*x*	The Speed of the Stylus, *v_os_*	Stylus Tip Radius, *r_tip_*	The Interval of Roughness Measurements	3D Topography Area
[mm]	[mm]	[-]	[-]	[μm]	[mm]	[μm]	[mm]	[mm]
0.8	4	100	1000	1	1	2	0.1	1.0 × 1.0

**Table 2:** The chemical composition of NiTi, $A_{f}=60\;℃$—the EDS (Energy-Dispersive Spectroscopy) analysis.

Element	wt. [%]	at. [*%*]
TiK	41.99	47.01
NiK	58.01	52.99
Total	100.00	100.00

**Table 3:** The physical, thermal, and mechanical properties of β-NiTi, $A_{f}=60\;℃$ [7,9].

Hardness	Thermal Conductivity	Tensile Strength, Ultimate	Tensile Strength, Yield	Modulus of Elasticity	Density	Structure (Phase)
[HV.1]	[W/m·°C]	[MPa]	[MPa]	[GPa]	[kg/m^3^]	
242	18	1364	649	28	6500	hi-temp B2

**Table 4:** Values of the cutting parameters $f_{z}$, $a_{e}$, and $v_{c}$ for NiTi alloy, selected by means of the RSM (response surface methodology).

No.	$\begin{array}{c} v_{c}\\ \left[m/min\right] \end{array}$	$\begin{array}{c} f_{z}\\ \left[mm/tooth\right] \end{array}$	$\begin{array}{c} a_{e}\\ \left[mm\right] \end{array}$
1	40	0.040	0.4
2	30	0.025	0.2
3	50	0.040	0.3
4	50	0.025	0.4
5	40	0.025	0.3
6	30	0.025	0.4
7	30	0.010	0.3
8	40	0.025	0.3
9	50	0.010	0.3
10	50	0.025	0.2
11	30	0.040	0.3
12	40	0.040	0.2
13	40	0.010	0.2
14	40	0.025	0.3
15	40	0.010	0.4

**Table 5:** Cutting force components according to the RSM.

No.	$\begin{array}{c} v_{c}\\ \left[m/min\right] \end{array}$	$\begin{array}{c} f_{z}\\ \left[mm/tooth\right] \end{array}$	$\begin{array}{c} a_{e}\\ \left[mm\right] \end{array}$	*F_y max_* = *F_f max_* [N]	*F_x max_* [N]	*F_t max_* [N]	*F_r max_* [N]
1	40	0.040	0.4	72.36	99.96	97.45	75.70
2	30	0.025	0.2	25.90	47.13	31.77	43.29
3	50	0.040	0.3	57.78	87.92	73.93	72.27
4	50	0.025	0.4	50.15	89.12	73.09	71.51
5	40	0.025	0.3	34.38	67.89	47.26	57.95
6	30	0.025	0.4	46.23	83.65	67.80	67.36
7	30	0.010	0.3	17.86	37.08	24.93	31.85
8	40	0.025	0.3	36.73	69.33	49.82	58.84
9	50	0.010	0.3	20.42	45.98	29.26	39.85
10	50	0.025	0.2	29.87	63.07	37.77	58.56
11	30	0.040	0.3	57.16	88.54	73.47	72.99
12	40	0.040	0.2	38.55	67.66	46.96	61.97
13	40	0.010	0.2	15.17	32.24	19.21	29.95
14	40	0.025	0.3	37.92	76.58	52.49	65.55
15	40	0.010	0.4	22.17	53.44	36.25	45.09

**Table 6:** Analysis of variance for cutting force components.

	Analysis of Variance for *F_rmax_*					
Source	DF	Seq SS	Adj SS	Adj MS	F	P
Regression	3	6591.84	6591.84	2197.28	74.16	0.000
Linear	3	6591.84	6591.84	2197.28	74.16	0.000
*v_c_* [m/min]	1	32.36	32.36	32.36	1.09	0.318
*f_z_* [mm/tooth]	1	4148.07	4148.07	4148.07	140.01	0.000
*a_e_* [mm]	1	2411.41	2411.41	2411.41	81.39	0.000
Residual Error	11	325.90	325.90	29.63		
Lack-of-Fit	9	312.26	312.26	34.70	5.09	0.175
Pure Error	2	13.64	13.64	6.82		
Total	14	6917.74				
	Analysis of Variance for *F_fmax_*					
Source	DF	Seq SS	Adj SS	Adj MS	F	P
Regression	3	3665.10	3665.10	1221.70	67.32	0.000
Linear	3	3665.10	3665.10	1221.70	67.32	0.000
*v_c_* [m/min]	1	15.32	15.32	15.32	0.84	0.378
*f_z_* [mm/tooth]	1	2821.13	2821.13	2821.13	155.46	0.000
*a_e_* [mm]	1	828.65	828.65	828.65	45.66	0.000
Residual Error	11	199.62	199.62	18.15		
Lack-of-Fit	9	193.13	193.13	21.46	6.61	0.138
Pure Error	2	6.49	6.49	3.25		
Total	14	3864.72				
	Analysis of Variance for *F_tmax_*					
Source	DF	Seq SS	Adj SS	Adj MS	F	P
Regression	3	2950.74	2950.74	983.58	36.24	0.000
Linear	3	2950.74	2950.74	983.58	36.24	0.000
*v_c_* [m/min]	1	89.11	89.11	89.11	3.28	0.097
*f_z_* [mm/tooth]	1	2318.73	2318.73	2318.73	85.43	0.000
*a_e_* [mm]	1	542.90	542.90	542.90	20.00	0.0001
Residual Error	11	298.55	298.55	27.14		
Lack-of-Fit	9	264.03	264.03	29.34	1.70	0.425
Pure Error	2	34.52	34.52	17.26		
Total	14	3249.28				

**Table 7:** Three-dimensional surface texture parameters according to ISO 25178.

No.	$\begin{array}{c} v_{c}\\ \left[m/min\right] \end{array}$	$f_{z}$ [mm/tooth]	$\begin{array}{c} a_{e}\\ \left[mm\right] \end{array}$	*Sa* [mm]	*Sz* [mm]	*Sq* [mm]	*Sp* [mm]	*Sv* [mm]	*Ssk* [-]	*Sku* [-]
1	40	0.040	0.4	1.08	11	1.37	6.14	4.83	0.02	3.22
2	30	0.025	0.2	1.02	15	1.44	11.8	3.21	2.01	10.50
3	50	0.040	0.3	1.11	14.9	1.55	10.9	3.95	1.56	7.49
4	50	0.025	0.4	2.13	25.8	3.16	9.58	16.2	−1.45	6.83
5	40	0.025	0.3	0.86	9.24	1.08	6.19	3.06	0.62	3.76
6	30	0.025	0.4	0.77	7.4	0.97	4.87	2.52	0.67	3.84
7	30	0.010	0.3	1.03	25.1	1.94	15.1	9.66	−0.04	4.00
8	40	0.025	0.3	0.75	6.36	0.91	3.3	3.06	−0.48	2.70
9	50	0.010	0.3	0.98	9.41	1.3	4.1	5.31	−0.35	3.77
10	50	0.025	0.2	0.84	5.28	1.05	2.27	3.01	−0.48	2.66
11	30	0.040	0.3	0.64	5.25	0.79	3.2	2.05	0.38	2.69
12	40	0.040	0.2	1.02	14.3	1.41	10.4	3.96	1.44	7.82
13	40	0.010	0.2	0.84	13.9	1.18	9.57	4.35	1.07	8.16
14	40	0.025	0.3	1.40	11.8	1.73	5.87	5.94	0.11	2.69
15	40	0.010	0.4	0.81	8.76	1.03	4.41	4.35	−0.11	3.18

**Table 8:** The appearance of the surface of different tests after milling (isometric images and Abbott curves).

**Test 11**: *v_c_* = 30 m/min; *f_z_* = 0.04 mm/tooth; *a_p_* = 0.3 mm		
*Sa* = 0.64 μm *Sz* = 5.25 μm *Sq* = 0.79 μm *Sp* = 3.2 μm *Sv* = 2.05 μm *Sku* = 2.69 *Ssk* = 0.38	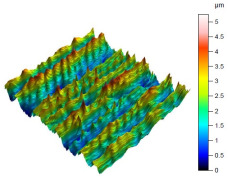	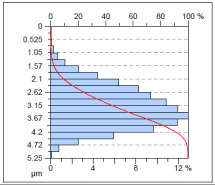
**Test 9**: *v_c_* = 50 m/min; *f_z_* = 0.01 mm/tooth; *a_p_* = 0.3 mm		
*Sa* = 0.98 μm *Sz* = 9.41 μm *Sq* = 1.3 μm *Sp* = 4.1 μm *Sv* = 5.31 μm *Sku* = 3.77 *Ssk* = −0.35	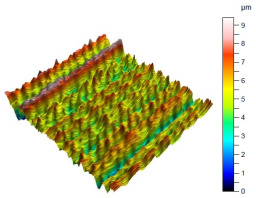	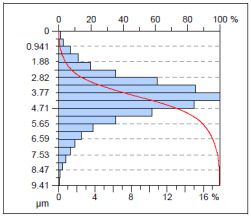
**Test 6:** *v_c_* = 30 m/min; *f_z_* = 0.025 mm/tooth; *a_p_* = 0.4 mm		
*Sa* = 0.77 μm *Sz* = 7.4 μm *Sq* = 0.97 μm *Sp* = 4.87 μm *Sv* = 2.52 μm *Sku* = 3.84 *Ssk* = 0.67	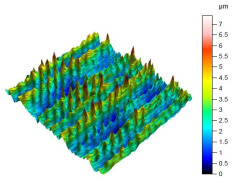	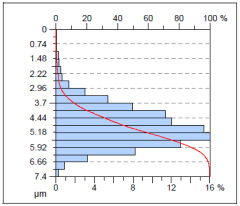
**Test 4:** *v_c_* = 50 m/min; *f_z_* = 0.025 mm/tooth; *a_p_* = 0.4 mm		
*Sa* = 2.13 μm *Sz* = 25.8 μm *Sq* = 3.16 μm *Sp* = 9.58 μm *Sv* = 16.2 μm *Sku* = 6.83 *Ssk* = −1.45	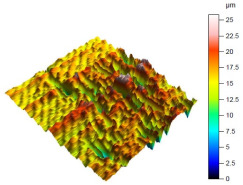	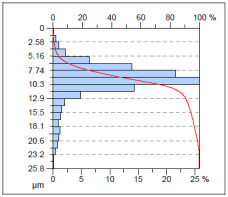
**Test 3:** *v_c_* = 50 m/min; *f_z_* = 0.04 mm/tooth; *a_p_* = 0.3 mm		
*Sa* = 1.11 μm *Sz* = 14.9 μm *Sq* = 0.79 μm *Sp* = 1.55 μm *Sv* = 3.95 μm *Sku* = 7.49 *Ssk* = 1.56	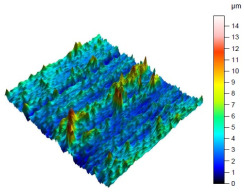	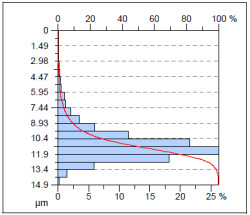
**Test 2:** *v_c_* = 30 m/min; *f_z_* = 0.025 mm/tooth; *a_p_* = 0.2 mm		
*Sa* = 1.02 μm *Sz* = 15.0 μm *Sq* = 1.44 μm *Sp* = 11.8 μm *Sv* = 3.21 μm *Sku* = 10.5 *Ssk* = 2.01	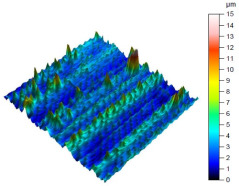	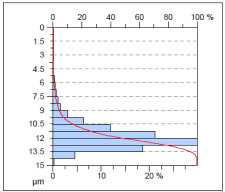

## Data Availability

Data are contained within the article.
